# Dietary Inflammatory Index and Depressive Symptoms in Chinese University Students Leveraging an Intelligent Ordering System: 3-Year Longitudinal Prospective Cohort Study

**DOI:** 10.2196/83784

**Published:** 2026-06-24

**Authors:** Peng Hong, Chen Hao, Zhou Weiqiang, Qian Jie, Zhang Yimeng, Ding Jingyun, Qian Haihong, Jia Yingnan

**Affiliations:** 1 Department of Preventive Medicine and Health Education School of Public Health Fudan University Shanghai China; 2 Informatization Office, Fudan University Shanghai China; 3 Mental Wellbeing Education and Counseling Center, Fudan University Shanghai China; 4 Institute of Health Communication, Fudan University, Shanghai 200032, China Shanghai China

**Keywords:** depressive symptoms, Dietary Inflammatory Index, intelligent ordering system, longitudinal prospective cohort study, university students

## Abstract

**Background:**

Depression is a major global cause of disability, and depressive symptoms are highly prevalent and increasing among Chinese university students. Mounting evidence confirms that inflammation plays a key role in the pathogenesis of depression, and dietary inflammatory potential regulates systemic inflammation to influence depressive symptom development. However, existing research is limited by cross-sectional designs, recall bias from self-reported dietary surveys, and a lack of long-term prospective cohort evidence on the diet-inflammation-mental health pathway in Chinese university students.

**Objective:**

This study aimed to examine the longitudinal association between Dietary Inflammatory Index (DII) and the incidence of depressive symptoms in Chinese university students, and to explore subgroup differences by family relationship and socioeconomic status.

**Methods:**

A 3-year longitudinal prospective cohort study was conducted among 5314 students from a university in Shanghai, China. Eligible participants met the criteria of ≥86 days of annual campus cafeteria dining and at least 1 breakfast, lunch, and dinner in campus canteens per quarter; students with abnormal monthly energy intake, excessive food consumption, or incomplete 3-year dietary/psychological data were excluded. Dietary data were continuously collected via the Intelligent Ordering System (IOS) from April 2020 to March 2023 to calculate DII scores. Depressive symptoms were annually assessed using the Beck Depression Inventory-II from March 2021 to March 2023. Mixed-effects logistic regression (α=.05) was used to analyze the association, with subgroup analyses stratified by family relationship and poverty status.

**Results:**

The baseline prevalence of depressive symptoms was 10.75% (571/5314; male: 261/2679, 9.74%; female: 310/2635, 11.76%). After adjusting for covariates, compared with the highest DII quartile (most proinflammatory diet), lower DII quartiles (more anti-inflammatory or low proinflammatory diets) were associated with a reduced risk of incident depressive symptoms in participants without depressive symptoms at baseline: Q1 (odds ratio [OR] 0.27, 95% CI 0.16-0.47), Q2 (OR 0.52, 95% CI 0.33-0.84), and Q3 (OR 0.26, 95% CI 0.16-0.42). Subgroup analyses showed this protective effect was only significant in students with harmonious family relationships and non–poverty-stricken students; no significant association between DII and depressive symptom improvement was found in participants with baseline depressive symptoms.

**Conclusions:**

This study is among the first to prospectively examine dietary inflammatory potential and depressive symptoms in university students using long-term objective dietary monitoring. Unlike studies relying on self-reported dietary surveys, this study used an automated and precise campus-based IOS to continuously capture real-world dietary behaviors over 3 years. The findings indicate that sustained anti-inflammatory dietary patterns are associated with a lower risk of depressive symptoms among Chinese university students, although this protective effect was weaker in students experiencing family discord or socioeconomic disadvantage. These findings provide new longitudinal evidence for the diet-inflammation-mental health relationship and support integrated campus interventions combining dietary guidance with psychosocial support.

## Introduction

### Problem

Depression is recognized as a multifaceted disorder that induces impairments in interpersonal, social, and occupational functioning [1]. The World Health Organization has indicated that depression ranks among the primary causes of disability globally, with projections showing it will significantly exacerbate the overall global disease burden by 2030 [2,3]. University life represents a pivotal transitional phase for students as they mature into adulthood, a period often accompanied by diverse challenges [4]. Students at this stage are particularly vulnerable to depression [5]. In the United States, an estimated 15.6% of undergraduate students exhibit symptoms of depression [6]. In China, the prevalence rate of depressive symptoms among college students has reached 24.71% and is continuously increasing [7]. Depression has risen from the eighth to the fourth leading cause of death among adolescents, with its associated disability-adjusted life years increasing from 2.8% to 3.7% [8]. A meta-analysis including 24 studies indicated that reported prevalence rates among college students ranged from 10% to 85%, with a weighted mean prevalence of 30.6% [9]. The presence of severe depressive symptoms not only leads to sleep disorders, poor concentration, anxiety, and apathy toward daily experiences but may also give rise to dangerous behaviors such as self-harm and suicidal ideation [9].

### Review of Relevant Scholarship

Growing robust evidence indicates that inflammation plays a pivotal role in the pathogenesis of depression, with individuals diagnosed with depression exhibiting elevated levels of multiple inflammatory markers, including C-reactive protein (CRP), interleukin-6, and tumor necrosis factor [10]. Diet, recognized as a critical modifiable lifestyle behavior, not only provides the energy and nutrients essential for sustaining life but also modulates systemic inflammatory levels through pathways such as inducing neuroinflammation and stimulating changes in oxidative stress, thereby influencing the development of depressive symptoms [11]. Healthy dietary habits, such as the Mediterranean diet pattern, typically include high amounts of vitamins, dietary fiber, polyphenolic phytochemicals, and unsaturated fatty acids, which exert anti-inflammatory and antidepressant effects [12,13]. By contrast, dietary factors that promote inflammation and depressive symptoms include oxidized lipids, saturated fatty acids, and trans fatty acids, which are highly prevalent in Western dietary patterns characterized by heavy consumption of sweets, refined grains, red and processed meats, snacks, and sugar-sweetened beverages [14].

Considering the complex composition of daily diets, which contain both proinflammatory and anti-inflammatory components, Shivappa et al [15] developed the Dietary Inflammatory Index (DII) to quantify dietary inflammatory levels by evaluating the effects of dietary components on 6 biomarkers: proinflammatory markers (CRP, interleukin-1β, interleukin-6, tumor necrosis factor-α) and anti-inflammatory markers (interleukin-4, interleukin-10) [16]. A lower DII score indicates a more anti-inflammatory diet, and a higher DII score reflects a more proinflammatory diet.

Previous studies have explored the association between dietary inflammatory levels and the risk of depressive symptoms among college students, but several limitations exist: first, bidirectional associations may exist between diet and depression; yet, a lack of cohort study evidence has hindered the clarification of the relationship between dietary inflammatory levels and depressive symptoms. Second, most studies predominantly rely on cross-sectional designs [17,18], which only investigate short-term dietary behaviors while overlooking the instability and malleability of college students’ dietary patterns, thus failing to adequately capture the long-term trends of dietary changes among this population. Third, several studies acquired data on college students’ food consumption through traditional survey methods, including 3-day recalls, 24-hour diet recalls, and food frequency questionnaire surveys, which introduced risks of recall bias [19].

### Hypothesis, Aims, and Objectives

The Intelligent Ordering System (IOS) has been operational at a university in Shanghai since 2020, providing long-term, continuous, and relatively accurate meal data for each diner. Its accuracy and effectiveness were verified in a prior study [20]. Our research used 3 consecutive years of dietary records (April 2020-March 2023) from the IOS to investigate the inflammatory potential of habitual diets on incident depressive symptoms, with subgroup analyses to clarify differential effects of dietary inflammation levels across distinct population strata.

## Methods

### Inclusion and Exclusion Criteria

Participants were selected if they had ≥86 days/year of school cafeteria dining attendance and consumed at least 1 breakfast, 1 lunch, and 1 dinner in campus canteens during each quarter. Individuals were excluded if they had abnormal monthly energy intake (male: <800 kcal/d or >4000 kcal/d; female: <500 kcal/d or >3500 kcal/d) or excessive food consumption (intake >1000 g/d). Students with incomplete 3-year dietary records or annual depressive symptom assessments were also excluded [20].

### Participant Characteristics

A total of 5314 university students from a university in Shanghai were included, covering undergraduates, master’s students, and doctoral candidates. All participants had valid 3-year dietary records from the IOS and completed annual depressive symptom assessments.

### Sampling Procedures

This study drew participants from the target population of undergraduate, master’s, and doctoral students at Fudan University. Sampling proceeded through 2 parallel data streams—dietary records and repeated psychological assessments—followed by data linkage to form the final analytic sample. For dietary data, records from the campus IOS were collected continuously over the study period. Sequential quality and completeness checks were applied to filter the raw dietary data, excluding cases that failed predefined criteria for data adequacy and validity, to yield a subset of students with reliable dietary assessment data. For psychological assessments, annual depressive symptom surveys were conducted at 3 time points. Starting with the baseline survey, follow-up assessments were conducted in subsequent years, with nonparticipants at each follow-up excluded from further analysis. This process identified students with complete 3-year psychological assessment data. The final analytic sample was formed by matching students from the valid dietary data subset with those from the complete psychological assessment subset, retaining only participants who met all data quality and completeness requirements for both streams throughout the study period.

### Sample Size, Power, and Precision

The intended sample size was determined by the availability of students with complete 3-year consecutive IOS dietary records and valid annual Beck Depression Inventory-II (BDI-II) assessments. The achieved sample size was 5314 participants, which was consistent with the planned sample derived from data completeness criteria. No a priori power analysis was performed, as this study was an observational cohort based on real-world objective dietary data; the large sample size ensured sufficient precision for estimating the association between DII and depressive symptoms. No interim analyses or stopping rules were used.

### Measures and Covariates

#### Acquiring Dietary Data From the IOS and Assessment of DII

We assessed daily food composition and nutrient intake on the basis of daily food purchase records from the IOS from April 2020 to March 2023. The IOS contains 5714 dishes, and we first established a food database by weighing the materials of 1840 representative dishes before and after cooking. For the remaining 3874 dishes, we used a standardized recipe approach and calculated their weights on the basis of the composition of the representative dishes. The database includes information on food composition, such as the weight of raw food materials and condiments, as well as the nutrient composition of each meal. Details of the weighing and calculation methods have been described in a previous paper [20]. Nutrient composition was determined via the Chinese Food Composition database (version 2023). Ultimately, we assessed the composition of 4 foods, including onion, ginger, garlic, and chili pepper and 20 nutrients, including energy, protein, fat, carbohydrate, fiber, cholesterol, vitamin A, carotene, thiamine (vitamin B1), riboflavin (vitamin B2), nicotinic acid (vitamin B3), vitamin C, vitamin E, magnesium (Mg), iron (Fe), zinc (Zn), selenium (Se), saturated fatty acid, monounsaturated fatty acid, and polyunsaturated fatty acid contents.

The DII is a literature-derived instrument used to quantify the inflammatory potential of the overall diet; lower DII scores indicate more anti-inflammatory dietary patterns, whereas higher scores indicate more proinflammatory dietary patterns [16,21]. In epidemiological studies, the DII is frequently analyzed in quantiles, particularly quartiles or quintiles, to compare outcome risks across different levels of dietary inflammatory potential, assess potential gradient or dose-response associations, and avoid relying solely on a linear assumption when DII is modeled as a continuous variable [22,23]. Therefore, in this study, DII was categorized into quartiles (Q1-Q4), with Q1 representing relatively lower dietary inflammatory potential (more anti-inflammatory dietary profiles) and Q4 representing relatively higher dietary inflammatory potential (more proinflammatory dietary profiles), to examine the association between different levels of dietary inflammatory potential and depressive symptoms [24]. We used the modified version of the DII calculation developed by Shivappa [16], and the specific calculation method has been elaborated in previous studies [18]. Among the theoretically possible list of 45 food parameters, we included the 4 types of foods and 20 nutrients mentioned earlier.

#### Assessment of Depressive Symptoms

We used the BDI-II [25] to assess depressive symptoms for 3 rounds (March 2021, 2022, and 2023). The BDI-II is a 21-item self-report scale that assesses the severity of depressive symptoms in the past 2 weeks and includes somatic-affective (13 items) and cognitive (8 items) dimensions. Each item is rated on a 4-point Likert scale ranging from 0 to 3, and total scores range from 0 to 63, with higher scores indicating more severe symptomatology. For this study, depression was dichotomized into no depressive symptoms (0-13) or prevalent depressive symptoms. The higher the total score is, the more severe the subject’s depression (a total score of 0-13 is considered nondepressed, 14-27 is considered mild depression, and 28-63 is considered moderate-severe depression) for Chinese young adults [26].

#### Covariates

We assessed covariates in 2021. The following measures were considered pertinent covariates because they are associated with depression and are independent risk factors for depression among young adults. For age, it was categorized into 2 groups: those younger than 23 years of age and those 23 years or older. Gender was divided into 2 groups: male and female. Poverty-stricken students were classified into 2 groups: yes and no. Living-school experience was grouped into having the experience and not having the experience. Only child was categorized as yes or no. Family relationships were divided into harmonious and disharmonious groups.

### Data Collection

Dietary data were automatically recorded in real time by the campus IOS from April 2020 to March 2023. Daily food composition and nutrient intake were derived from IOS meal records, combined with a standardized dish database. Depressive symptoms were assessed annually via self-administered BDI-II questionnaires in March 2021, 2022, and 2023.

### Quality of Measurements

For dietary intake measurement, data were automatically and objectively recorded by the campus IOS, which eliminated recall bias. Standardized procedures for dish weighing, recipe calculation, and nutrient composition derivation were applied to ensure the accuracy and consistency of dietary data.

For depressive symptom measurement, the BDI-II was administered as a standardized self-report questionnaire with uniform instructions to all participants. To enhance data quality and prevent careless or random responding, common-sense check items and quality control questions were embedded in the questionnaire. Invalid questionnaires identified by these quality control items were excluded before data analysis, ensuring the reliability and validity of the outcome measurement.

### Instrumentation

IOS: a campus-based automated platform with 5714 dishes; a food database was established by weighing 1840 representative dishes, with nutrient values derived from the Chinese Food Composition Database (2023). The validity of IOS for dietary assessment has been verified in prior studies.

BDI-II: a 21-item self-report scale measuring depressive symptom severity in the past 2 weeks, scored 0-3 per item (total 0-63).

### Masking

No masking (blinding) was implemented in this study. Participants, data collectors, and outcome assessors were not blind to group assignments or exposure status, as this was an observational cohort study without experimental manipulation.

### Psychometrics

The BDI-II has been validated in Chinese young adult populations with acceptable reliability and validity. For this sample, no additional reliability analysis was performed; the psychometric properties of BDI-II used in this study were based on published validation data for Chinese populations.

### Conditions and Design

This study used a nonexperimental longitudinal observational cohort design without experimental manipulation. Participants were observed naturally over 3 years, with repeated assessments of dietary inflammatory potential and depressive symptoms.

### Data Diagnostics

Missing data: the study database contained no missing values; no imputation methods were needed.

Outlier exclusion: predefined criteria for energy intake and food consumption outliers were applied before data analysis (as listed in the Inclusion and Exclusion Criteria section).

Data distribution: no data transformations were performed; statistical assumptions were evaluated during regression analysis.

### Analytic Strategy

The characteristics of the participants and the quartile groups of the DII are presented as frequencies with percentages. A chi-square test was conducted to explore whether there were differences in the distribution of the DII quartile groups and depressive symptoms among different demographic characteristics at baseline. Mixed-effects logistic regression was used to assess the impact of different DII levels on the incidence of depressive symptoms, and odds ratios (ORs) and their 95% CIs were calculated. Individual-level random intercepts were incorporated into the model to capture unobserved heterogeneity between participants and appropriately handle within-subject correlation. Time was explicitly modeled as a categorical variable (year) to account for temporal variations in depression status. The model included DII quartile groups as the primary exposure variable, with the highest quartile (most proinflammatory diet) serving as the reference group. The variables of age group, family relationship, gender, and whether being a poverty-stricken student showed significant differences in distribution during the baseline demographic tests. Regarding the impact of DII levels on depressive symptom occurrence, for these variables, we performed further subgroup analyses using mixed-effects logistic regression to examine if there were differences in each subgroup. The database used in this study contains no missing values, and thus, no handling of missing data was involved. All statistical analyses were performed using R software (version 4.1.1; R Foundation for Statistical Computing) and Python (version 3.7; Python Software Foundation).

### Ethical Considerations

This study was conducted in accordance with the Declaration of Helsinki and was reviewed and approved by the Ethics Committee of Medical Research, School of Public Health, Fudan University (IRB#2019-01-0726 S; approval date: January 7, 2019), with no exemptions from human subject research ethics oversight. Written informed consent was obtained from all participants prior to data collection, and the original informed consent documents approved by the institutional review board explicitly permitted secondary analysis of the anonymized dietary and psychological data without requiring additional consent from participants. All study data were anonymized and deidentified prior to analysis, with no personal identifiers (eg, name, student ID, and contact information) retained to protect participant privacy and confidentiality. No financial, material, or other forms of compensation were provided to participants for their involvement in this study. No images or materials containing personally identifiable information of individual participants are included in the manuscript or supplementary materials, so no additional consent for the use of identifiable images is required.

## Results

### Participant Flow

**Figure 1:**
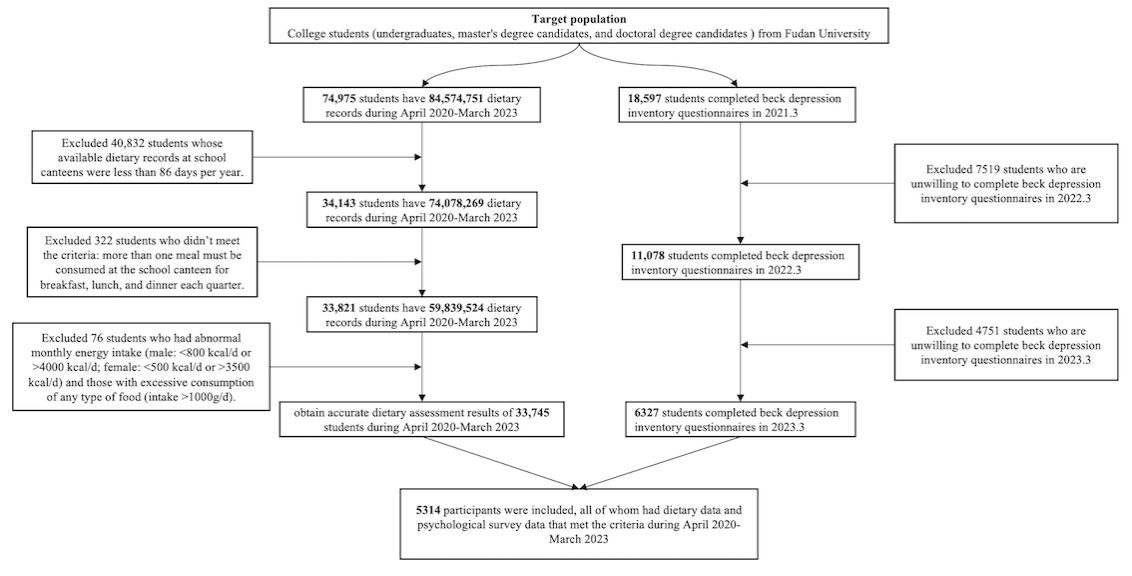
Flowchart of participant enrollment and follow-up in a 3-year longitudinal prospective cohort study of dietary inflammatory index and depressive symptoms among university students in Shanghai, China (April 2020-March 2023).

Participant flow through the study is presented in Figure 1.

### Recruitment

Recruitment and repeated assessments were conducted across a 3-year period. Dietary data were continuously collected via the IOS from April 2020 to March 2023, with semester breaks (January, February, July, and August of each year) excluded from analyses. Depressive symptoms were assessed annually at 3 fixed time points: March 2021 (baseline), March 2022 (first follow-up), and March 2023 (second follow-up). All participants were enrolled from a single university in Shanghai.

### Statistics and Data Analysis

As shown in Table 1, we included 5314 young adults, of whom 2728 (51.34%) were female, 2586 (48.66%) were male, 3422 (64.4%) were aged between 17 and 23 years, 1892 (35.6%) were aged 23 years or older, 640 (12.04%) had poor relationships between their family, 824 (15.51%) were poverty-stricken students, 3201 (60.24%) had residential experience, and 3327 (62.61%) were only child.

**Table 1:** Baseline characteristics of participants in a 3-year longitudinal prospective cohort study of Dietary Inflammatory Index and depressive symptoms among university students in Shanghai, China (March 2021).

Baseline characteristics		Overall (n=5314), n (%)	Nondepressive (n=4743), n (%)	Depressive (n=571), n (%)	*P* value
**Gender**					.17
	Female	2635 (49.59)	2325 (88.24)	310 (11.76)	
	Male	2679 (50.41)	2418 (90.26)	261 (9.74)	
**Age group**					.01
	17-23 years	1892 (35.60)	1716 (90.7)	176 (9.3)	
	Over 23 years	3422 (64.40)	3027 (88.46)	395 (11.54)	
**Family relationship**					<.001
	Disharmonious	640 (12.04)	483 (75.47)	157 (24.53)	
	Harmonious	4674 (87.96)	4260 (91.14)	414 (8.86)	
**Poverty-stricken students**					<.001
	No	4490 (84.49)	4040 (89.98)	450 (10.02)	
	Yes	824 (15.51)	703 (85.32)	121 (14.68)	
**Living-school experience**					.50
	No	2113 (39.76)	1894 (89.64)	219 (10.36)	
	Yes	3201 (60.24)	2849 (89)	352 (11)	
**Only** **child**					.17
	No	1987 (37.39)	1758 (88.48)	229 (11.52)	
	Yes	3327 (62.61)	2985 (89.72)	342 (10.28)	

At baseline, young adults exhibited a median depression score of 4.0 (IQR 0-9.0) on the BDI-II, with 571 participants meeting criteria for depressive symptoms and 4743 remaining symptom-free. During the follow-up period, among the 4743 participants without depressive symptoms at baseline, 451 (9.5%) experienced the onset of depressive symptoms; among the 571 participants with depressive symptoms at baseline, depressive symptoms disappeared in 318 (55.6%). Table 1 also describes the distribution of several characteristics according to depressive symptoms. Students aged 23 years and older have a higher proportion of experiencing depressive symptoms compared to those <23 years of age. College students with disharmonious family relationships and those from poverty-stricken backgrounds have a higher proportion of experiencing depressive symptoms.

The median score at baseline study was 0.36 (IQR –2.23 to 1.97), which indicated a slightly proinflammatory diet. Table 2 illustrates the baseline characteristics by DII quartiles in the sample as a whole. Gender distribution varied markedly among quartiles (*P*=.004), with a slight but notable increase in female representation in Q4 (proinflammatory diet: 663/2635, 25.16%) compared to Q1 (anti-inflammatory diet: 654/2635, 24.82%). Poverty-stricken status exhibited a distinct nonlinear pattern (*P*=.04), peaking in Q3 (low-grade proinflammatory diet: 221/824, 26.82%) before declining sharply in Q4 (190/824, 23.06%).

**Table 2:** Baseline characteristics of university students by quartiles of Dietary Inflammatory Index in a 3-year longitudinal prospective cohort study in Shanghai, China (April 2020-March 2023).

Baseline characteristics				Q1 (n=1329), n (%)	Q2 (n=1328), n (%)	Q3 (n=1328), n (%)	Q4 (n=1329), n (%)	*P* value	
**Gender**									.004
		Female		654 (24.82)	706 (26.79)	612 (23.23)	663 (25.16)		
		Male		675 (25.20)	622 (23.22)	716 (26.73)	666 (24.86)		
**Age group**									.45
			≥23 years	450 (23.78)	474 (25.05)	487 (25.74)	481 (25.42)		
			<23 years	879 (25.69)	854 (24.96)	841 (24.58)	848 (24.78)		
**Family relationship**									.75
	Disharmonious			158 (24.69)	151 (23.59)	162 (25.31)	169 (26.41)		
	Harmonious			1171 (25.05)	1177 (25.18)	1166 (24.95)	1160 (24.82)		
**Poverty-stricken students**									.04
		No		1144 (25.48)	1100 (24.50)	1107 (24.65)	1139 (25.37)		
		Yes		185 (22.45)	228 (27.67)	221 (26.82)	190 (23.06)		
**Living-school experience**									.06
	No			533 (25.22)	487 (23.05)	548 (25.93)	545 (25.79)		
	Yes			796 (24.87)	841 (26.27)	780 (24.37)	784 (24.49)		
**Only child**									.40
	No			477 (24.01)	519 (26.12)	494 (24.86)	497 (25.01)		
	Yes			852 (25.61)	809 (24.32)	834 (25.07)	832 (25.01)		

As shown in Table 3, among participants with depressive symptoms at baseline, no statistically significant associations were observed. Among participants without depressive symptoms at baseline, Model 2, which adjusted for baseline covariates, showed that compared with those in the proinflammatory diet group (Q4), participants in the other 3 groups with lower dietary inflammatory levels had significantly lower odds of incident depressive symptoms: Q1 (OR 0.27, 95% CI 0.16-0.47), Q2 (OR 0.52, 95% CI 0.33-0.84), and Q3 (OR 0.26, 95% CI 0.16-0.42).

**Table 3:** Association between Dietary Inflammatory Index (DII) and incident depressive symptoms among university students in a 3-year longitudinal prospective cohort study in Shanghai, China (April 2020-March 2023).

Groups and DII		Model 1^a^		Model 2^b^	
		OR^c^ (95% CI)	*P* value	OR (95% CI)	*P* value
**Baseline participants with depressive symptoms**					
	Q1	1.00 (0.77-1.32)	.97	0.81 (0.55-1.19)	.28
	Q2	0.95 (0.72-1.26)	.74	0.70 (0.49-1.06)	.05
	Q3	1.01 (0.77-1.34)	.92	0.86 (0.63-1.18)	.36
	Q4	Reference	N/A^d^	Reference	N/A
**Baseline participants without depressive symptoms**					
	Q1	1.23 (0.75-2.03)	.41	0.27 (0.16-0.47)	<.001
	Q2	0.94 (0.57-1.57)	.82	0.52 (0.33-0.84)	.007
	Q3	1.19 (0.74-1.93)	.47	0.26 (0.16-0.42)	<.001
	Q4	Reference	N/A	Reference	N/A

^a^Model 1 was unadjusted for any covariates.

^b^Model 2 adjusted for covariates at baseline: gender, age group, family relationship, poverty-stricken students, living-school experience, and only child.

^c^OR: odds ratio.

^d^N/A: not applicable.

As shown in Table 4, among participants with depressive symptoms at baseline, we did not find any significant association between dietary inflammatory potential and improvement in depressive symptoms.

**Table 4:** Subgroup analysis of the association between Dietary Inflammatory Index (DII) and depressive symptom improvement among baseline depressed students in a 3-year longitudinal prospective cohort study in Shanghai, China (April 2020-March 2023).

Character and DII				Model 1^a^				Model 2^b^			
				OR^c^ (95% CI)		*P* value		OR (95% CI)		*P* value	
**Family relationship**											
	**Harmonious (n=414)**										
		Q1	1.03 (0.76-1.41)		.84		1.31 (0.74-2.30)		.36		
		Q2	1.00 (0.73-1.38)		.98		1.01 (0.56-1.81)		.97		
		Q3	1.06 (0.77-1.47)		.71		1.49 (0.85-2.59)		.16		
		Q4	Reference		N/A^d^		Reference		N/A		
	**Disharmonious (n=157)**										
		Q1	0.83 (0.44-1.57)		.57		0.98 (0.37-2.58)		.96		
		Q2	0.74 (0.39-1.42)		.37		0.71 (0.27-1.84)		.48		
		Q3	0.72 (0.39-1.32)		.29		0.71 (0.29-1.72)		.45		
		Q4	Reference		N/A		Reference		N/A		
**Poverty-stricken students**											
	**Yes** **(** **n=121** **)**										
		Q1	0.78 (0.43-1.40)		.40		0.98 (0.43-2.24)		.96		
		Q2	0.89 (0.48-1.64)		.71		0.76 (0.32-1.77)		.52		
		Q3	1.18 (0.65-2.15)		.59		1.53 (0.70-3.35)		.29		
		Q4	Reference		N/A		Reference		N/A		
	**No (n=450)**										
		Q1	1.08 (0.79-1.46)		.64		1.32 (0.75-2.35)		.34		
		Q2	0.98 (0.71-1.34)		.88		1.02 (0.57-1.83)		.94		
		Q3	0.97 (0.71-1.34)		.87		1.07 (0.62-1.85)		.81		
		Q4	Reference		N/A		Reference		N/A		

^a^Model 1 was unadjusted for any covariates.

^b^In the family relationship subgroup, Model 2 adjusted for covariates at baseline: gender, age group, poverty-stricken students, living-school experience, and only child; in the poverty-stricken student subgroup, Model 2 adjusted for covariates at baseline: gender, age group, family relationship, living-school experience, and only child.

^c^OR: odds ratio.

^d^N/A: not applicable.

As shown in Table 5, among participants without depressive symptoms at baseline, Model 2 showed particularly pronounced reductions in the odds of depressive symptoms among those with harmonious family relationships: Q1 (OR 0.38, 95% CI 0.25-0.59), Q2 (OR 0.28, 95% CI 0.20-0.40), and Q3 (OR 0.49, 95% CI 0.36-0.66), compared with the proinflammatory diet group (Q4). In contrast, no significant associations were observed among participants with disharmonious family relationships in either model.

**Table 5:** Subgroup analysis of the association between Dietary Inflammatory Index (DII) and incident depressive symptoms among baseline nondepressed students in a 3-year longitudinal prospective cohort study in Shanghai, China (April 2020-March 2023).

Character and DII				Model 1^a^					Model 2^b^	
				OR^c^ (95% CI)		*P* value		OR (95% CI)		*P* value
**Family relationship**										
	**Harmonious (n=4260)**									
		Q1	0.64 (0.40-1.02)		.06		0.16 (0.07-0.33)			<.001
		Q2	0.68 (0.45-1.02)		.06		0.24 (0.13-0.46)			<.001
		Q3	0.80 (0.55-1.16)		.25		0.56 (0.34-0.91)			.02
		Q4	Reference		N/A^d^		Reference			N/A
	**Disharmonious (n=483)**									
		Q1	1.36 (0.83-2.22)		.22		1.16 (0.62-2.16)			.64
		Q2	0.85 (0.50-1.42)		.53		0.65 (0.33-1.29)			.22
		Q3	1.20 (0.74-1.94)		.46		1.02 (0.55-1.88)			.96
		Q4	Reference		N/A		Reference			N/A
**Poverty-stricken students**										
	**Yes (n=703)**									
		Q1	0.87 (0.50-2.05)		.98		1.00 (0.49-2.04)			>.99
		Q2	0.83 (0.56-1.95)		.88		1.05 (0.56-1.95)			.88
		Q3	1.04 (0.61-2.09)		.69		1.13 (0.61-2.09)			.69
		Q4	Reference		N/A		Reference			N/A
	**No (n=4040)**									
		Q1	0.74 (0.48-1.14)		.17		1.51 (0.86-2.64)			.15
		Q2	0.60 (0.40-0.89)		.01		0.25 (0.13-0.49)			<.001
		Q3	0.76 (0.53-1.08)		.13		1.42 (0.86-2.33)			.17
		Q4	Reference		N/A		Reference			N/A

^a^Model 1 was unadjusted for any covariates.

^b^In the family relationship subgroup, Model 2 adjusted for covariates at baseline: gender, age group, poverty-stricken students, living-school experience, and only child; in the poverty-stricken student subgroup, Model 2 adjusted for covariates at baseline: gender, age group, family relationship, living-school experience, and only child.

^c^OR: odds ratio.

^d^N/A: not applicable.

When stratified by poverty status, both models showed protective associations between anti-inflammatory dietary profiles and a lower risk of depressive symptoms exclusively among non–poverty-stricken students. In Model 1, the association was observed for Q2 compared with Q4 (OR 0.60, 95% CI 0.40-0.89); this pattern persisted in Model 2 (OR 0.25, 95% CI 0.13-0.49). No statistically significant differences across DII quartiles were observed among poverty-stricken students.

## Discussion

### Support of Original Hypotheses

To our best knowledge, this is the first study to conduct long-term dietary monitoring and prospective analysis of the association between dietary inflammatory potential and the incidence of depressive symptoms among the college student population [27,28]. The study used a longitudinal cohort design involving 5314 university students, integrating objective dietary assessments via IOS—a campus-based platform for automated dietary consumption data recording—with annual depressive symptom evaluations using the BDI-II. In participants without depressive symptoms at baseline, our longitudinal evidence finds an association between dietary inflammatory potential and depressive symptom incidence, demonstrating that adherence to anti-inflammatory or low proinflammatory diets was associated with a reduced risk of having depressive symptoms. Notably, this protective effect exhibited substantial subgroup heterogeneity. Individuals experiencing socioeconomic disadvantages or familial discordance derived fewer benefits from anti-inflammatory dietary patterns, highlighting the moderating role of psychosocial contexts in these relationships. In participants with depressive symptoms at baseline, we did not find any significant association between dietary inflammatory potential and depressive symptom improvement.

### Similarity of Results

In this study, the prevalence of depressive symptoms among university students was 10.75% (571/5314), with rates of 11.76% (310/2635) in female and 9.74% (261/2679) in male individuals. These figures are substantially lower than reported prevalence rates among university students in other global regions (Europe: 24.1%, North America: 35.5%, Africa: 40.1%, Asia: 34.8%) [29]. In China, university students aged <23 years are predominantly undergraduates, while those >23 years of age are mainly postgraduates [30]. This study found significantly higher prevalence of depressive symptoms among university students aged 23 years or older compared to their younger counterparts, indicating elevated risk among graduate students. Previous studies have identified graduate students as a high-risk population for various mental health problems, with contributing factors including academic pressures, financial strain, stress, and career uncertainty [31,32]. These cumulative stressors have established depression as both a prevalent psychological disorder in this population and 1 occurring at 6 times the rate observed in the general population [33]. At baseline, the prevalence of depressive symptoms was significantly higher in the family relationship disharmonious group than in the harmonious group and among poverty-stricken students compared to non–poverty-stricken students. Previous studies have consistently demonstrated that family warmth, cohesion, and supportive parenting practices are associated with improved mental health outcomes and reduced depressive symptoms in adolescents [34]. Furthermore, a study from Hubei Province, China, revealed that childhood exposure to emotional abuse, domestic violence, and physical neglect increased the risk of developing depressive symptoms in adulthood by 80%, 88%, and 39%, respectively [35]. A UK-based study found that university students from affluent families had a 70% lower probability of developing depressive symptoms [36], which aligns with our findings. Students with higher socioeconomic status benefit from greater access to mental health services, academic support resources, and stable living environments—all of which help mitigate psychological stress and promote better mental health outcomes [37].

Regarding dietary inflammatory potential, we observed a significantly higher proportion of proinflammatory diets among male compared to female individuals, consistent with numerous previous studies [38]. This disparity likely stems from gender-specific dietary preferences: male individuals tend to consume greater quantities of proinflammatory foods such as red meat and processed meats, while female individuals show stronger preferences for anti-inflammatory dietary components, including vegetables, whole grains, tofu, and high-cocoa dark chocolate [39]. In our study, non–poverty-stricken students demonstrated relatively even distribution across all 4 quartiles (Q1-Q4) of dietary inflammatory potential, whereas poverty-stricken students showed greater concentration in both mildly proinflammatory (Q3) and mildly anti-inflammatory diets (Q2), with fewer individuals in anti-inflammatory diets (Q1) and proinflammatory diets (Q4) categories. This finding contrasts with previous research indicating a positive association between socioeconomic status and proinflammatory diets [40]. The observed discrepancy may stem from unique pricing characteristics of university dining halls; more expensive menu items tend to be highly processed, richer in oil, salt, and sugar, and predominantly feature red meat, while nutritionally balanced, minimally processed options are substantially cheaper [41-43]. Consequently, non–poverty-stricken students can easily maintain balanced dietary patterns, resulting in higher proportions in Q2 and Q3 [44].

Compared to university students with proinflammatory diets (Q4), those exhibiting anti-inflammatory (Q1 and Q2) or low proinflammatory (Q3) dietary patterns demonstrated reduced risks of depressive symptoms. Our findings partially align with previous studies conducted in Iranian, Turkish, Spanish, and Australian populations. In Iranian adolescents, consumption of a proinflammatory diet, reflected by higher DII scores, was associated with significantly greater odds of exhibiting at least moderate depressive symptoms compared to female individuals consuming the least anti-inflammatory diets (OR 3.96, 95% CI 1.12-13.97) [45]. A Turkish study examined students in the nutrition and dietetics department of a private university’s faculty of health sciences in Istanbul. It identified a positive association between DII and depressive symptoms (OR ranging between 1.64-1.67), supporting that the inflammatory burden of diet correlates with students’ propensity for depression [17]. A Spanish study involving 15,093 university graduates analyzed the relationship between dietary inflammatory potential and depression risk, finding that participants in the highest DII quintile (most proinflammatory diet) had a 47% higher depression risk compared to those in the lowest quintile (hazard ratio 1.47, 95% CI 1.17-1.85), demonstrating a significant dose-response relationship [46]. A 9-year cohort study using the middle-aged cohort of the Australian Longitudinal Study on Women’s Health, which included a total of 6438 women, found that women with the most anti-inflammatory diet had an approximately 20% lower risk of developing depression compared with women with the most proinflammatory diet (relative risk 0.81, 95% CI 0.69, 0.96), suggesting that an anti-inflammatory diet is associated with a lower risk of depression in middle-aged Australian women [15].

### Interpretation

Numerous studies across diverse populations have demonstrated analogous findings [47]. This pattern is also broadly consistent with evidence in university students, among whom better overall diet quality has generally been associated with better mental health, whereas stress and anxiety have often been linked to poorer diet quality, suggesting that the potential mental health benefits of healthier eating may be harder to realize under sustained psychosocial strain [48]. However, in our subgroup analyses, the same dietary behavior did not significantly reduce the risk of depressive symptom occurrence among students categorized as family relationship disharmonious and poverty-stricken. At baseline, the prevalence of depressive symptoms was significantly higher in the family relationship disharmonious group and poverty-stricken students, suggesting an inherent vulnerability to depressive symptoms in populations exhibiting these characteristics [49,50]. Mechanistically, parental conflicts, marital problems, or harsh punishment that create adverse family environments immediately increase depression risk through psychological distress [51]. Beyond these proximal psychological effects, early adversity can shape the hypothalamic-pituitary-adrenocortical axis and other stress-response systems, producing more persistent neuroendocrine dysregulation that may amplify inflammatory signaling and vulnerability to later psychopathology [52,53]. Chronically, such environments stimulate proinflammatory cytokine production, elevating serum CRP and interleukin-6 [54]. Similarly, adverse economic conditions lead to a series of detrimental sequelae, including prolonged psychological distress, inadequate nutritional intake, and accelerated biological aging processes [55], further elevating systemic inflammation [54]. In young adults, economic hardship is also closely intertwined with food insecurity, which has been repeatedly associated with poorer mental health, sleep disturbance, and depressive symptoms, and may reduce both the quality and consistency of anti-inflammatory food intake [52,56,57]. The Pittsburgh Girls Study established that greater family adversity during ages 5-9 years predicted higher interleukin-6 levels in adolescence (β=.14; *P*=.048), with persistence into early adulthood (β=.12; *P*=.001) [58]. Childhood financial strain independently predicted adult interleukin-6 elevations (β=.09; *P*=.009) [59]. Prior research confirms childhood socioeconomic disadvantage and family discordance promote chronic inflammation through biological embedding mechanisms, including dysregulated stress response systems and adiposity-driven inflammatory pathways [60,61]. Conversely, close and supportive family relationships have been conceptualized as resilience factors that can buffer the biological embedding of adversity and contribute to more favorable long-term health profiles [62]. Consequently, even after implementing anti-inflammatory or low proinflammatory diets, systemic inflammation may remain elevated, thereby diminishing dietary interventions’ capacity to reduce depression risk.

### Strengths and Limitations

This study has several key strengths. First, its 3-year longitudinal cohort design enabled follow-up of dietary behaviors and depressive symptoms, supporting a temporal association not possible with prior cross-sectional research. Second, the IOS-collected canteen meal data were recorded automatically and continuously, ensuring objectivity and accuracy. Third, subgroup analyses revealed nuanced interactions between dietary inflammation and psychosocial factors, showing that anti-inflammatory diets’ protective effects are shaped by familial and socioeconomic contexts—with direct implications for targeted interventions.

There are several limitations in this study. First, the meal data from the IOS cannot provide a comprehensive overview of all food consumed by individuals daily. Although validation studies confirmed that campus dining records reflect students’ primary dietary patterns, unmeasured consumption of off-campus foods (eg, fruits, beverages, and snacks) may have introduced nondifferential misclassification in DII calculations, potentially attenuating observed associations. Second, constraints in covariate breadth—particularly the absence of data on physical activity, sleep quality, and substance use—precluded full adjustment for potential behavioral confounders. Third, the inclusion criteria requiring complete 3-meal records from campus dining halls may have introduced selection bias by excluding students with irregular eating habits. Admittedly, students with more irregular dietary behaviors are more likely to experience adverse depressive symptoms. Nevertheless, after careful consideration, we retained these inclusion and exclusion criteria to ensure the representativeness of participants’ dietary behaviors in the study.

### Conclusion

This study is among the first to prospectively examine dietary inflammatory potential and depressive symptoms in university students using long-term objective dietary monitoring. Unlike previous studies relying mainly on self-reported dietary surveys, this study used an automated and precise campus-based IOS to continuously capture real-world dietary behaviors over 3 years. The findings indicate that sustained anti-inflammatory dietary patterns are associated with a lower risk of depressive symptoms among Chinese university students, although this protective effect was weaker in students experiencing family discord or socioeconomic disadvantage. These findings provide new longitudinal evidence for the diet-inflammation-mental health relationship and support integrated campus interventions combining dietary guidance with psychosocial support.

## Data Availability

The data presented in this study are available upon request from the corresponding author. The data are not publicly available due to the privacy of the study participants.

## Abbreviations

BDI-II: Beck Depression Inventory-II
CRP: C-reactive protein
DII: Dietary Inflammatory Index
IOS: Intelligent Ordering System
OR: odds ratio
